# An inflammation-related signature could predict the prognosis of patients with kidney renal clear cell carcinoma

**DOI:** 10.3389/fgene.2022.866696

**Published:** 2022-08-11

**Authors:** Qingxin Yu, Facai Zhang, Dechao Feng, Dengxiong Li, Yuhui Xia, Mei-Fu Gan

**Affiliations:** ^1^ Department of Pathology, Taizhou Hospital, Wenzhou Medical University, Linhai, China; ^2^ Department of Urology, Institute of Urology, West China Hospital, Sichuan University, Chengdu, China

**Keywords:** inflammation, renal clear cell carcinoma, immune infiltration, signature, immune checkpoint

## Abstract

**Background:** Kidney renal clear cell carcinoma (KIRC) is an inflammation-related carcinoma, and inflammation has been recognized as an important factor in inducing carcinogenesis. To further explore the role of inflammation in KIRC, we developed an inflammation-related signature and verified its correlation with the tumor micro-environment.

**Methods:** After the differential inflammation-related prognostic genes were screened by Lasso regression, the inflammation-related signature (IRS) was constructed based on the risk score of multivariate Cox regression. Then, the prognostic value of the IRS was evaluated by Kaplan-Meier analysis, receiver operating characteristic (ROC) curve analysis and multivariate Cox regression. Gene set variation analysis (GSVA) was applied to screen out enriched signaling pathways. Infiltrated immune cells, tumor mutational burden (TMB) and immune checkpoints were explored by CIBERSORTx and maftool.

**Results:** Four genes (TIMP1, PLAUR, CCL22, and IL15RA) were used to construct the IRS in patients with KIRC. Kaplan-Meier analysis and multivariate Cox regression identified that the IRS could independently predict the prognosis of patients with KIRC in the training and validation groups. The diagnostic value of the nomogram increased from 0.811 to 0.845 after adding the IRS to the multiparameter ROC analysis. The GSVA results indicated that IRS was closely related to primary immunodeficiency and antigen processing and presentation. The immune checkpoint LAG3 was highly expressed in patients with high-risk score (*p* < 0.05), while CD274 (PD-L1) and HAVCR2 were highly expressed in patients with low-risk score (*p* < 0.001). There was a significant positive correlation between the high-risk score group and CD8^+^ T, activated CD4^+^ memory T, gamma and delta regulatory T and M0 macrophage cells, while the low-risk score group was negatively associated with B memory, plasma, resting CD4^+^ memory T, activated NK, M1 macrophages and resting mast cells.

**Conclusion:** We found that the IRS might serve as a biomarker to predict the survival of KIRC. Moreover, patients with high or low-risk score might be sensitive to immune drugs at different immune checkpoints.

## Introduction

Worldwide, there were 431,288 newly diagnosed kidney cancers in 2020, which represented approximately 2.2% of all cancers (Sung et al., 2021). In United States, according to the data of 2022, the incidence of kidney cancer occupied sixth and ninth in the new cancers of male and female, respectively (Siegel et al., 2022). Of these, renal cell carcinoma (RCC) accounts for approximately 90% of all kidney malignancies. The main pathological type of RCC is kidney renal clear cell carcinoma (KIRC), which accounts for 70%–80% of cases (Nerich et al., 2014). Partial and radical nephrectomy is the optimal therapeutic choice for located KIRC. Unfortunately, approximately one-third of patients with localized RCC inevitably develop metastases, which need systemic treatment to control the disease (Escudier et al., 2019). Given the poor therapeutic outcome, chemotherapy was used to cure patients with KIRC. In recent years, to further improve the prognosis of KIRC, immunotherapy has been applied clinically (Escudier et al., 2019). However, few biomarkers can precisely predict the prognosis and therapeutic outcome of KIRC, which hinders the personalized application of these therapies and the creation of new drugs. This dilemma prompted us to explore the potential mechanism of the occurrence and progression of KIRC.

Chronic inflammation has been recognized as an important factor for carcinogenesis by inducing oxidative and nitrative DNA damage (Ohnishi et al., 2013). Moreover, inflammatory cells are a major component of the tumor microenvironment and an indispensable factor in promoting tumor proliferation, neoplastic processes, survival, and migration (Okada et al., 2021). Tumor-associated inflammation has been widely studied and listed as a hallmark of cancers (Hanahan, 2022). Moreover, Zhao et al. (2019) identified five immune- and inflammation-related core clusters by integrating multiomics data to identify the role of immunity and inflammation in KIRC. To further explore the role of inflammation in KIRC, Marona et al. (2017) identified that low MCPIP1 levels could increase proliferation, tumor outgrowth, and vascularity by upregulating inflammation by degrading mRNAs encoding proinflammatory cytokines in KIRC. Clinically, an included 10-study meta-analysis identified that the systemic immune-inflammation index could independently predict survival outcomes in patients with renal cell carcinoma. KIRC is an inflammation-related carcinoma, which inspired us to clarify the role of inflammation in KIRC. And these evidences suggest that we should construct an inflammation-related signature (IRS) and validate whether it can be used as a potential biomarker for KIRC.

Therefore, this study first constructed an IRS for patients with KIRC and validated the prognostic value of this signature. Furthermore, we explored the correlation between the IRS and immunotherapy by evaluating the tumor mutational burden, immune checkpoint and immune cell infiltration in KIRC.

## Materials and methods

### Data collection

The clinical and RNA_seq data of KIRC were downloaded from Cancer Genome Atlas (www.gdc.cancer.gov, TCGA) (Wei et al., 2018) database. After excluding patients with postoperative survival times shorter than 30 days, the remaining patients were randomly divided into the TCGA training group (*n* = 364) and TCGA test groups (*n* = 156). Then, the TCGA training group was used to develop the IRS, while the TCGA test group was employed to validate the signature. GSE29609 (Edeline et al., 2012) was extracted from the Gene Expression Omnibus (Edgar et al., 2002) to further estimate the diagnostic and prognostic value of the IRS. Before analysis these clinical and RNA_seq data, the removeBatchEffect function in limma package was used to reduce the batch effects of the TCGA and GEO datasets.

### Identification of differentially expressed inflammatory genes and biological functional analysis

To identify the differentially expressed genes, the mRNA data of 520 KIRC samples and 72 normal samples were compared by the “limma” package with False Discover 99 Rate (FDR) < 0.05. The inflammation-related genes were provided by hallmark genes of the Molecular Signature Database (Subramanian et al., 2005). In detail, the inflammation-related gene set was generated by a computational methodology based on identifying gene set overlaps and retaining genes that display coordinate expression. Moreover, the Molecular Signatures Databases team provided the microarray data that served for refining and validation of the inflammation-related gene set online (Liberzon et al., 2015). Furthermore, the “Venn Diagram” package in R software was used to screen out the co-expressed inflammation-related genes in the TCGA dataset and GSE29609.

To explore possible biological functions and signaling pathways, the “cluster Profiler” package in R software was used to analyze all differentially expressed genes. In Gene Ontology (GO) enrichment analysis, the results with *p*-value < 0.05 and q-value < 0.05 were collected and visualized in bar plots based on molecular function (MF), biological process (BP) and cellular component (CC) categories. Similarly, according to the results of Kyoto Encyclopedia of Genes and Genomes (KEGG) analysis, the signaling pathways with *p*-value < 0.05 and q-value < 0.05 were included and visualized in a bubble plot. Based on a linear regression-based algorithm and a label propagation algorithm, GeneMania (www.genemania.org) (Warde-Farley et al., 2010) was employed to predict interacting genes and generate a visual figure.

### Development and validation of an inflammation-related signature

To identify a stable and predictive inflammation-related signature, a lasso regression model was employed to evaluate all differentially expressed inflammation-related genes. In this process, the penalty parameter (*λ*) of the model was controlled by 10-fold cross-validation. Referring to the lasso results, the IRS was constructed based on the selected genes. Furthermore, risk score was generated in a multivariate Cox regression model with the following formula:

$$\mathrm{Risk}\;\mathrm{score}=\sum_{i=1}^{n}\mathrm{coefficien}t_{i}*\mathrm{EXP}{\left(mRNA\right)}_{i}$$ (1)

All patients were divided into high- and low-risk groups according to the median risk scores. To validate the prognostic value of the IRS, Kaplan-Meier analysis was performed to compare the survival time and outcomes between the high- and low-risk score KIRC patients in the TCGA training group, TCGA test group and GSE29609 group.

### Correlation of the inflammatory signature with clinical parameters

To further validate the relationship between the IRS and clinical parameters, subgroups were generated by classifying age, sex, T stage, metastasis, and AJCC stage and comparing the different risk score in these subgroups. To assess the discernibility of the IRS, we performed principal component analysis and depicted the risk score plot depending on the risk score and survival outcome. The Wilcoxon rank-sum test was used to compare the risk score in each subgroup. Kaplan-Meier analysis was performed to estimate the prognosis of different risk score in each subgroup. Receiver operating characteristic (ROC) curve analysis was used to assess the diagnostic ability of the IRS in the TCGA training, TCGA test and GSE29609 groups. Furthermore, the independent prognostic ability of the IRS and clinical parameters was evaluated by a multivariate Cox regression model.

### Gene set variation analysis, tumor mutational burden analysis, and immune cell infiltration

Gene Set Variation Analysis (GSVA) (Hänzelmann et al., 2013) was performed by the “GSVA” package in R. The GSVA results were generated by comparing the high- and low-risk score groups and visualized by heatmap. We uploaded the transcriptome data of high- and low-risk score patients in TCGA training and a collection of KEGG pathways from the Molecular Signature Database (MSigDB, version 7.4) (Liberzon et al., 2015) for GSVA. For the estimation of tumor mutational burden (TMB) in KIRC, tumor mutational burden analysis used the “maftools” package to calculate the mutation rate of each sample downloaded from the TCGA database. After calculating the mutation rate, the samples were divided into high and low TMB groups according to the TMB score, and the survival time and outcome between the groups were compared by Kaplan-Meier analysis.

TMB and immune checkpoint-associated mismatched repair genes in tumor tissue were considered potential biomarkers for predicting immunotherapy response. Thus, we compared the transcriptome data of CD274 (PD-L1), PDCD1LG2 (PD-L2), CTLA4, HAVCR2, LAG3, PDCD1, TIGIT, and SIGLEC15 in both risk score groups. Furthermore, after normalization of transcriptome gene expression data of KIRC patients with the “limma” package, the CIBERSORT algorithm was utilized to evaluate the immune infiltration of 22 leukocyte subtypes (LM22), which were downloaded from the known reference set on the CIBERSORTx website (Newman et al., 2015). The infiltration difference between the high- and low-risk score groups was calculated with the Wilcoxon rank-sum test, and a boxplot was used for visualization. Meanwhile, Tumor Immune Dysfunction and Exclusion (TIDE) algorithm, acquiring data from the TCGA-KIRC cohort, was used to predict and compare the immunotherapy response of patients in high- or low-risk score group (Jiang et al., 2018; Fu et al., 2020).

### Development and validation of a predictive nomogram

To quantitatively predict the prognosis of KIRC patients, a nomogram was carefully established based on the risk score and clinical parameters, including age, M stage and AJCC stage. Of these, patients over 60 years old were divided into elderly group and the remaining young group. For the purpose of validation, the concordance index (C-index), multiparameter ROC analysis, decision curve analysis (DCA) and calibration curves were used to validate the reliability and accuracy of the nomogram.

### Statistical analysis

All analyses were carried out using R software, version 4.1.2. Quantitative data in two groups were compared using Student’s *t* test. Quantitative data were compared with one-way analysis of variance (ANOVA) or Welch’s test in three or more groups. *P* < 0.05 was regarded as statistically significant.

## Results

### Patient characteristics, co-expressed inflammation genes and biological functional analysis

**FIGURE 1:**
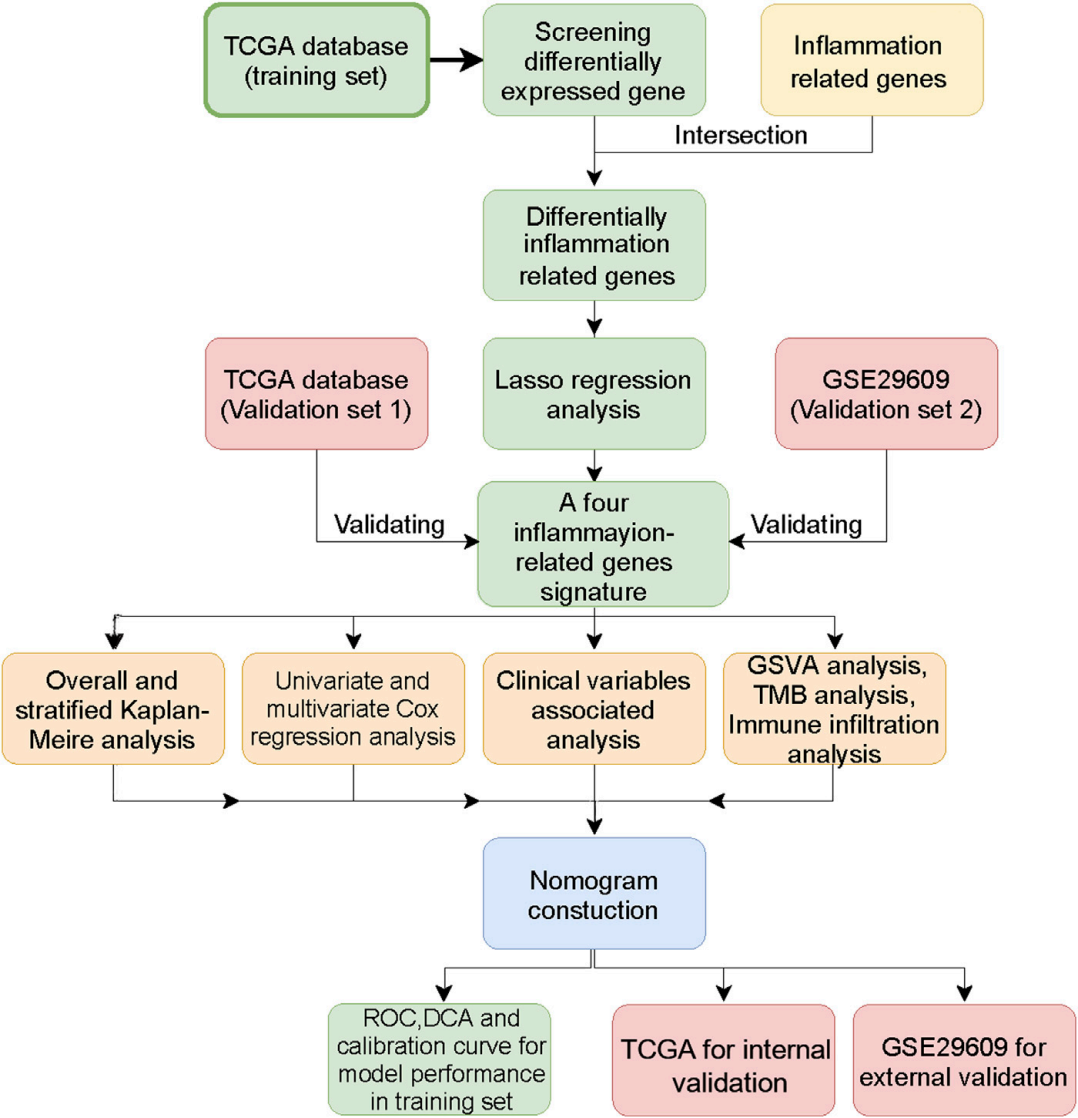
The workflow of this study.

**FIGURE 2:**
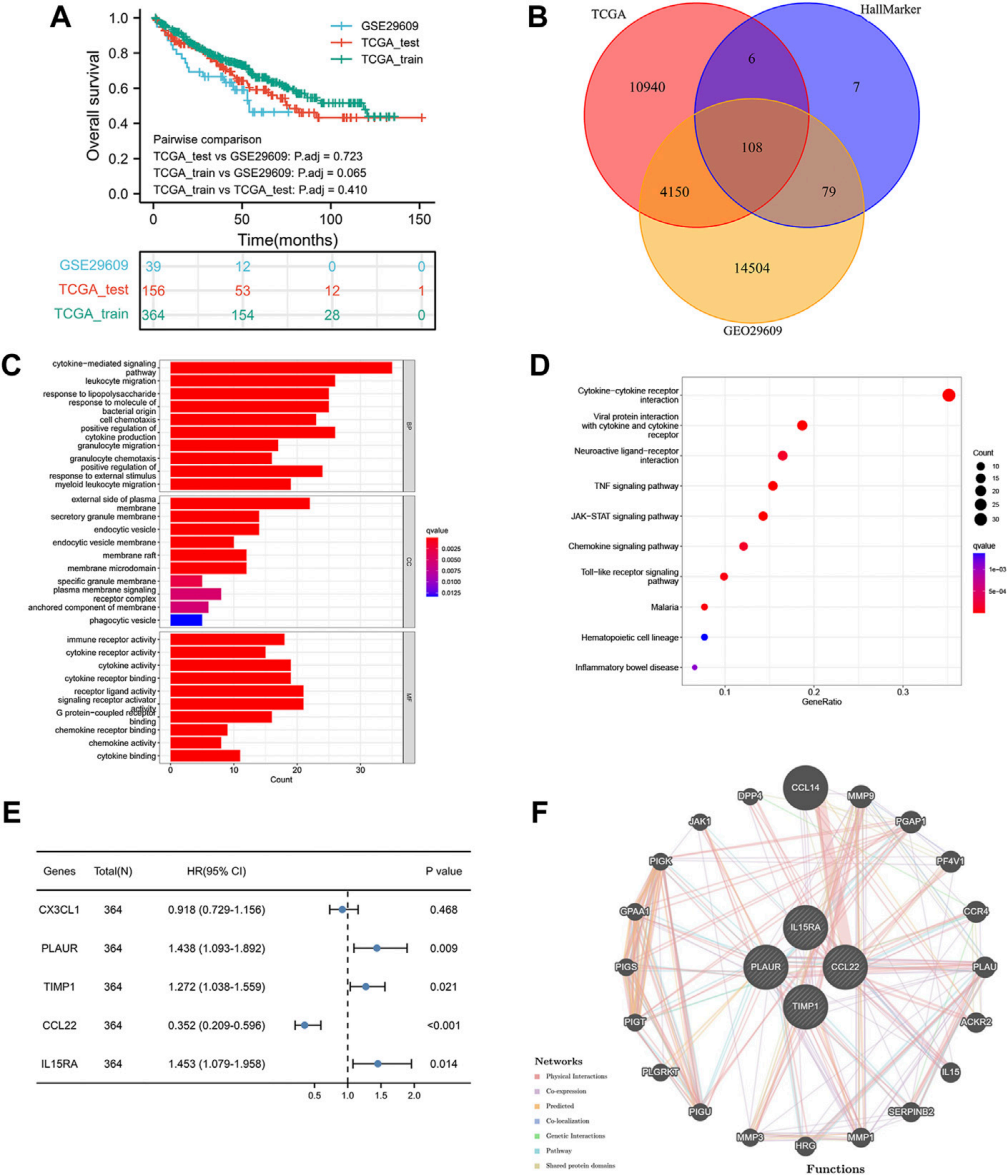
Construction of inflammation-related signature: the overall survival of included patients **(A)**, the Venn diagram of differentially coexpressed genes **(B)**, the GO result **(C)**, the KEGG result **(D)**, the results of five genes in multivariate Cox regression model **(E)**, the result of protein–protein interaction network **(F)**.

Figure 1 shows the workflow of our work. Table 1 presents the clinicopathologic features of all included patients. There were 364, 156, and 39 patients included in the TCGA training, TCGA test and GSE29609 groups, respectively. Figure 2A provides the survival outcomes of the TCGA-train, TCGA-test and GSE29609 groups, and no significant difference existed among these three groups. A total of 15,453 differentially expressed genes were collected with the absolute value of the log2-transformed fold change (FC) > 1 and the adjusted *p* value (adj. *p*) < 0.05 was used as the threshold after normalization and batch effect removal. Then, 108 co-expressed inflammatory genes were selected by Venn Diagram (Figure 2B). Further GO analysis showed that the co-expressed inflammatory genes were positively correlated with cytokine-mediated signaling pathways, leukocyte migration, the external side of plasma and immune receptor activity (Figure 2C). The results of KEGG analysis showed that the co-expressed inflammatory genes were positively associated with cytokine–cytokine receptor interactions (Figure 2D).

**TABLE 1:** Clinicopathologic characteristics of the included patients.

Characteristic	GSE29609	TCGA test	TCGA train
n	39	156	364
Age, n (%)			
Older	17 (3%)	50 (8.9%)	127 (22.7%)
Young	22 (3.9%)	106 (19%)	237 (42.4%)
Sex, n (%)			
female	NA	60 (11.5%)	117 (22.5%)
Male	NA	96 (18.5%)	247 (47.5%)
T stage, n (%)			
1	11 (2%)	74 (13.2%)	193 (34.5%)
2	5 (0.9%)	25 (4.5%)	45 (8.1%)
3	22 (3.9%)	54 (9.7%)	118 (21.1%)
4	1 (0.2%)	3 (0.5%)	8 (1.4%)
M stage, n (%)			
M0	25 (4.7%)	115 (21.5%)	302 (56.6%)
M1	14 (2.6%)	30 (5.6%)	48 (9%)
AJCC stage, n (%)			
Stage I	15 (2.7%)	72 (12.9%)	189 (33.9%)
Stage II	3 (0.5%)	21 (3.8%)	37 (6.6%)
Stage III	13 (2.3%)	32 (5.7%)	85 (15.3%)
Stage IV	8 (1.4%)	30 (5.4%)	52 (9.3%)
Survival status, n (%)			
Alive	22 (3.9%)	99 (17.7%)	250 (44.7%)
Dead	17 (3%)	57 (10.2%)	114 (20.4%)

TCGA, cancer genome atlas; n, number; NA, no data; M, metastasis; AJCC, american joint committee on Cancer.

As shown in Figure 2E, the lasso regression model selected five inflammatory genes (CX3CL1, TIMP1, PLAUR, CCL22, and IL15RA). To assess the independent prognostic value of these genes, a multivariate Cox regression model was used to calculate the five genes. The results illustrated that TIMP1, PLAUR, CCL22, and IL15RA could independently predict the prognosis of patients with KIRC (*p* < 0.05). According to the GeneMANIA results, we found that TIMP1, PLAUR, CCL22, and IL15RA proteins were associated with some proteins, such as CCL14, PF4V1, ACKR2, and PGAP1 (Figure 2F).

### Establishment and validation of the inflammation-related gene signature

**FIGURE 3:**
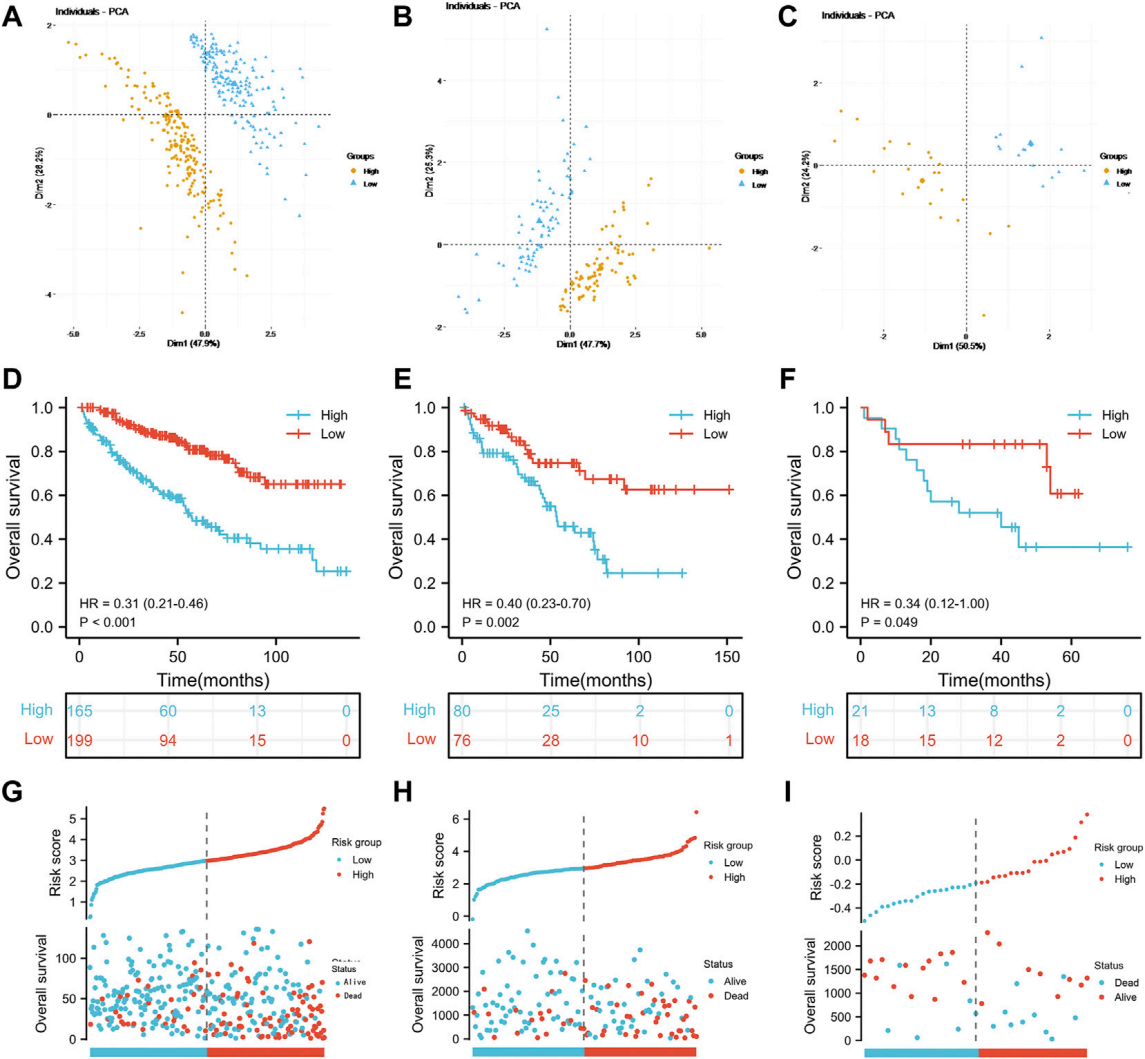
Prognostic performance of the inflammation-related signature: Principal component analysis results of the TCGA training group **(A)**, TCGA test **(B)** and GSE29609 **(C)**. Kaplan-Meier analysis results of the TCGA training group **(D)**, TCGA test **(E)** and GSE29609 **(F)**. The risk score plots of TCGA training group **(G)**, TCGA test **(H)** and GSE29609 **(I)**.

Referring to the results of the multivariate Cox regression model above, TIMP1, PLAUR, CCL22 and IL15RA were used to construct the IRS. Patients in the three groups were divided into the high-risk score group and the low-risk score group according to the median risk score of the TCGA training group, TCGA test group and GSE29609 group. As shown in Figures 3A–C, the risk scores clearly differed between the high- and low-risk score groups in the TCGA training group, TCGA test group and GSE29609 group. There were significant differences in overall survival (OS) between the high- and low-risk score groups in the TCGA training group (Figure 3D, *p* < 0.001). Similarly, patients in the high-risk score group of TCGA-test group and GSE29609 group were correlated with worse OS than those in the low-risk group, even GSE29609 provided only 39 patients (Figures 3E,F, *p* = 0.002, *p* = 0.049, respectively). Furthermore, a positive correlation was found between the increased IRS score and the increase in mortality in the TCGA training group, TCGA test group and GSE29609 group (Figures 3G–I).

### Correlation between the inflammation-related signature and clinical parameters

**FIGURE 4:**
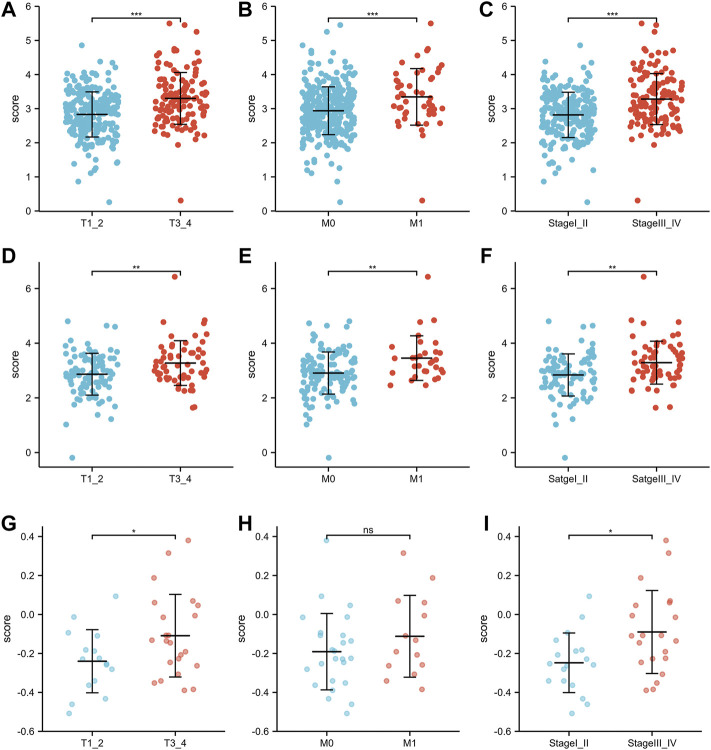
The risk scores of patients in different clinicopathological parameters: TCGA training group: T stage **(A)**, M stage **(B)**, AJCC stage **(C)**. TCGA-test group: T stage **(D)**, M stage **(E)**, AJCC stage **(F)**. GSE29609: T stage **(G)**, M stage **(H)**, AJCC stage **(I)**.

As shown in Figures 4A–C, there were significant differences in the risk scores at T stage, M stage and AJCC stage in the TCGA training group (all *p* < 0.001). Similarly, in the TCGA-test group, patients with T3-4 or M1 or AJCC stage III-IV were associated with higher risk scores than those with low stage (Figures 4D–F, all *p* < 0.01). In the GSE29609 group, patients with T3-4 or AJCC stage III-IV were correlated with higher risk scores than patients with low stage, but not M1 (Figures 4G–I).

**FIGURE 5:**
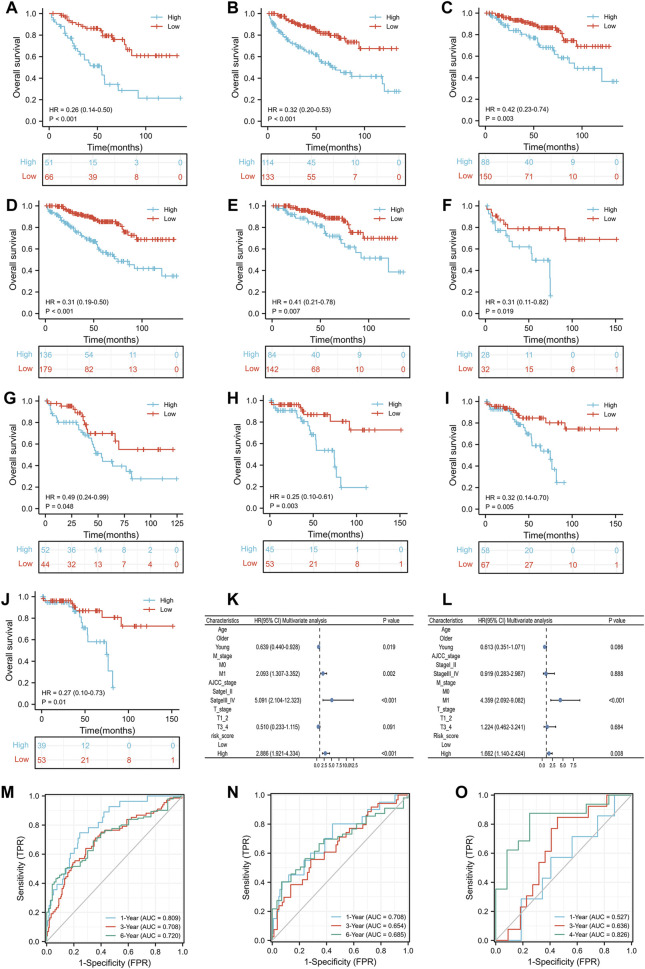
The prognostic ability of the risk scores: Kaplan-Meier analysis results of subgroups in TCGA training group: female **(A)**, male **(B)**, T1_2 **(C)**, M0 **(D)** and AJCC stage I-II **(E)**; Kaplan-Meier analysis results of subgroups in TCGA test group: female **(F)**, male **(G)**, T1_2 **(H)**, M0 **(I)** and AJCC stage I_II **(J)**; Results of multivariate Cox regression model for parameters in TCGA training group **(K)**; Results of multivariate Cox regression model for parameters in TCGA test group **(L)**; Receiver operating characteristic curve analysis results of TCGA training group **(M)**, TCGA test group **(N)** and GSE29609 **(O)**.

Prior to analyzing the prognosis of the subgroups, we reported that the IRS score could predict the prognosis of the TCGA training group, TCGA test group and GSE29609 group in the previous section. In the subgroup analysis, high-risk score patients in the TCGA training group were associated with worse OS than those with low-risk scores, including females (Figure 5A, *p* < 0.001), males (Figure 5B, *p* < 0.001), T1-2 (Figure 5C, *p* = 0.003), M0 (Figure 5D, *p* < 0.001), and AJCC stage I-II (Figure 5E, *p* = 0.007). The results of the subgroup analysis in the TCGA-test group were similar to those in the TCGA-training group; patients with high-risk scores were correlated with shorter OS than patients with low-risk scores, including females (Figure 5F, *p* = 0.019), males (Figure 5G, *p* = 0.048), T1-2 (Figure 5H, *p* = 0.003), M0 (Figure 5I, *p* = 0.005), and AJCC stage I-II (Figure 5J, *p* = 0.01). We did not perform Kaplan-Meier analysis in GSE29609 because of the limited number of patients. Figure 5K illustrates that age, M stage, AJCC stage and IRS score were independent predictors of patients in the TCGA training group. To further validate the predictive value of the IRS, we performed a multivariate Cox regression model in the TCGA-test group and identified that M1 stage and high-risk scores were independently associated with worse OS (Figure 5L). As Figure 5M shows, the areas under the curve (AUCs) of the IRS score in the TCGA training group were 0.809, 0.708 and 0.720 at 1, 3, and 6 years, respectively. In the TCGA-test group, the AUC of the IRS score was 0.708, 0.654, and 0.685 at 1, 3, and 6 years, respectively (Figure 5N). Due to the limited number of patients in GSE29609, we only calculated the AUCs at 1, 3, and 4 years, which were 0.527, 0.636, and 0.826, respectively (Figure 5O).

### Gene set variation analysis, tumor mutational burden analysis, and immune cell infiltration

**FIGURE 6:**
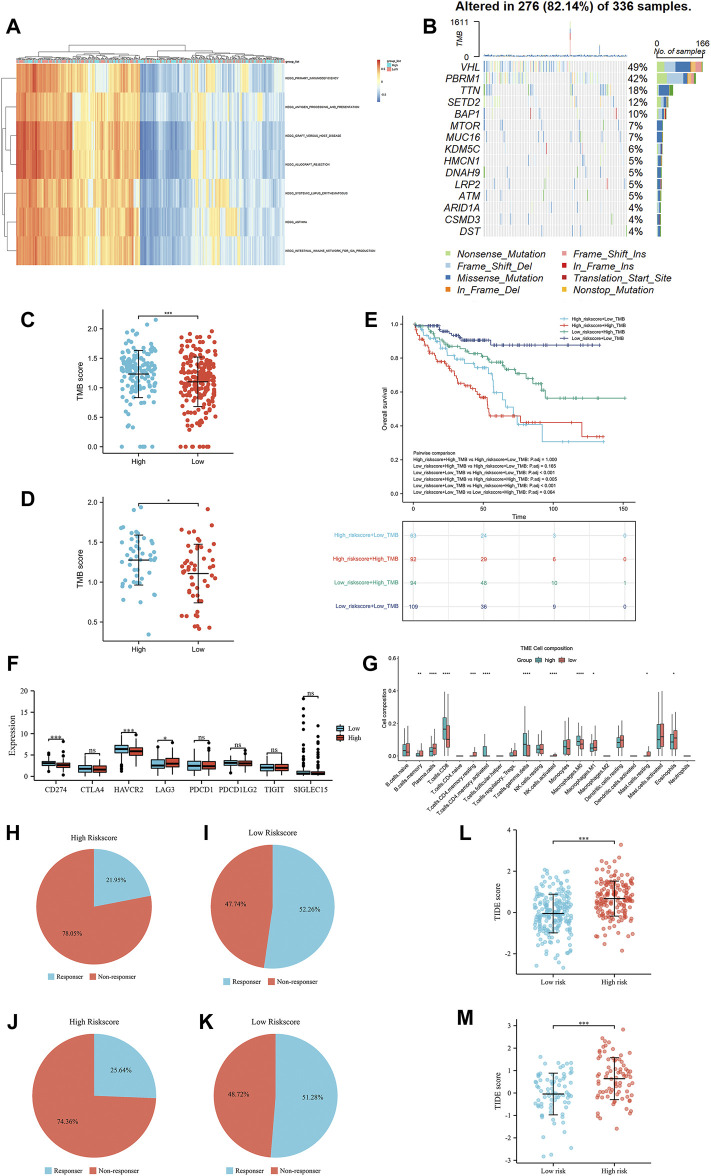
The results of gene set variation analysis **(A)**, the results of tumor mutational burden analysis **(B)**, the correlation of tumor mutational burden scores and the risk scores in the TCGA training group **(C)**, the correlation of tumor mutational burden scores and the risk scores in the TCGA test group **(D)**, the OS of the high-risk group compared with that in the low-risk group in the TCGA training group **(E)**, the expression of immune checkpoints in the high-risk group compared with that in the low-risk group in the TCGA training group **(F)**, and the immune cell infiltration in the high-risk group compared with that in the low-risk group in the TCGA training group **(G)**. The TIDE immunotherapy response outcome of high-risk score group **(H)** and low-risk score group **(I)** in the TCGA training group, high-risk score group **(J)** and low-risk score group **(K)** in the TCGA test group. The TIDE score of the TCGA training group **(L)** and the TCGA test group **(M)**.

As shown in Figure 6A, the enrichment pathways included primary immunodeficiency, antigen processing and presentation and systemic lupus erythematosus, which were mainly correlated with inflammation and immune pathways.

The waterfall presented the top 15 most mutated genes, VHL and PBRM1, making up the majority of the mutations (Figure 6B). Of these, the majority of mutations were nonsense mutations, missense mutations and frame shift del. To further assess the prognostic value of the TMB score, all patients with TMB scores were divided into four groups according to the TMB score and IRS score. The TMB in the high-risk-score group was significantly higher than that in the low-risk-score group in the TCGA training and TCGA test groups (Figures 6C,D). The OS of these four groups was analyzed by Kaplan-Meier analysis, which showed that patients with low-risk scores and low TMB scores were associated with a better OS than patients in other groups (Figure 6E). Interestingly, LAG3 expression in the high-risk score group was higher than that in the low-risk score group, while the expression of mismatch repair genes CD274 (PD-L1) and HAVCR2 in the high-risk score group was lower than that in the low-risk score group (Figure 6F). The Wilcoxon rank-sum test displayed a remarkable discrepancy between the high- and low-risk score groups in 22 immune cell types. There was a significant positive correlation between the high-risk score group and CD8^+^ T, activated CD4^+^ memory T, gamma and delta regulatory T and M0 macrophage cells, while the low-risk score group was negatively associated with B memory, plasma, resting CD4^+^ memory T, activated NK, M1 macrophages and resting mast cells (Figure 6G). Referring the results of TIDE, patients with low-risk score in TCGA training group and test group had higher immunotherapy response rate than patients with high-risk score (Figures 6H–K). Similarly, patients with low-risk score were associated with lower TIDE score than patients with low-risk score (Figures 6L,M).

### Development and validation of a predictive nomogram

**FIGURE 7:**
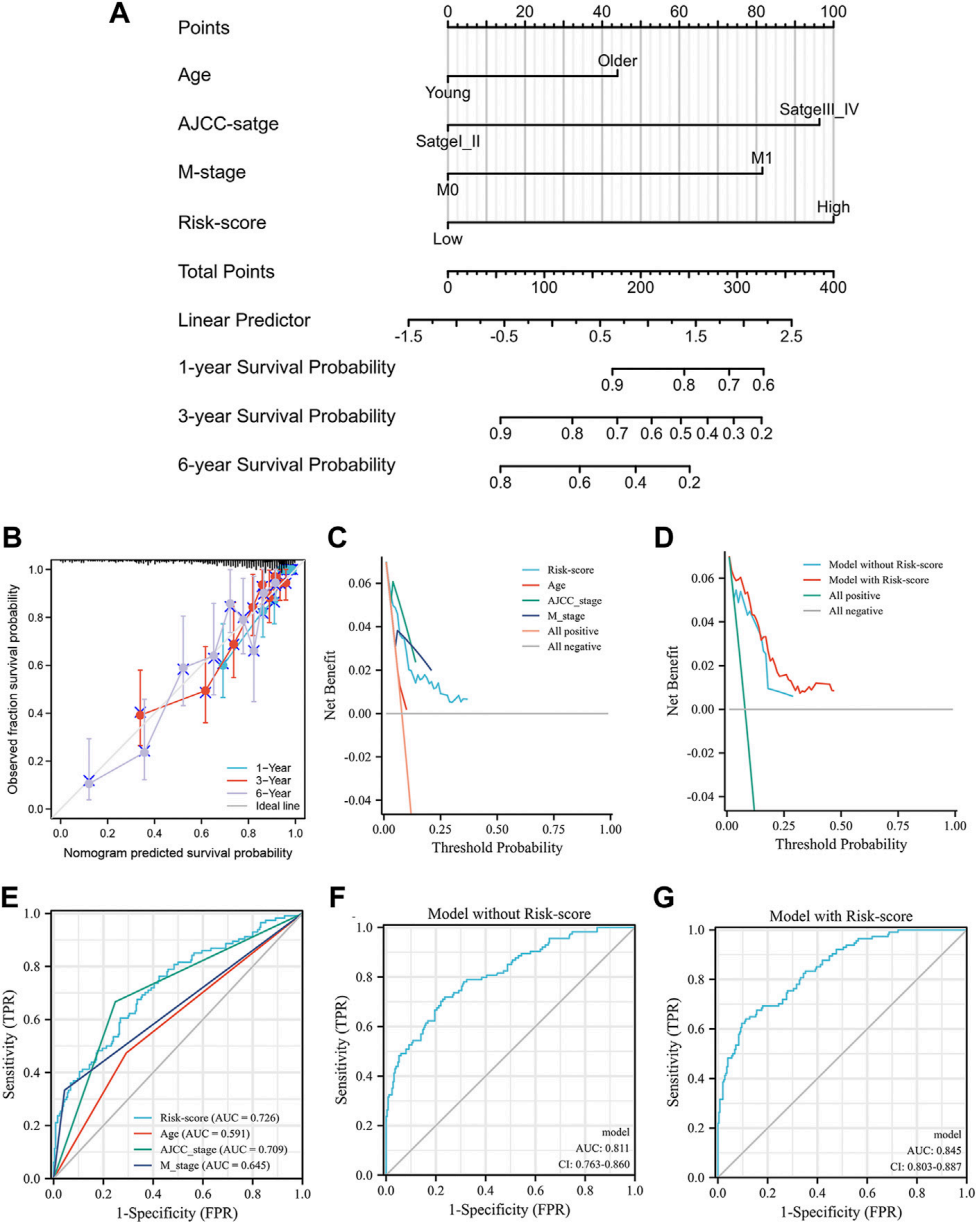
The nomogram of the inflammation-related gene signature and its performance: the nomogram **(A)**, the calibration curves of the nomogram **(B)**, the calibration curves of different factors **(C)** and the nomogram **(D)**, the result of ROC analysis with different factors **(E)**, the result of multiparameter ROC analysis without the risk scores **(F)**, the result of multiparameter ROC analysis with the risk scores **(G)**.

The correlation between the prognosis of KIRC, the IRS score and clinical parameters was tested using a multivariate Cox regression model based on the TCGA training group in the previous section (Figure 5K). The parameters with *p* < 0.05 were selected for further analysis according to the results of the multivariate Cox regression model. To extend the clinical applicability of the IRS, a nomogram containing parameters with age, AJCC stage, IRS score and M stage was constructed based on the TCGA training group (Figure 7A). The concordance index of this module was 0.784 (0.762–0.805). The calibration curves at 1, 3, and 6 years showed that there was good agreement between the predicted value and the true value (Figure 7B). Interestingly, the consistency between the predicted value and the true value increased with time. Furthermore, compared with clinical parameters, the IRS score exhibited potential clinical value (Figure 7C). This nomogram was superior to either the “all positive” or “all negative” model in predicting the prognosis of KIRC (Figure 7D). In detail, module with IRS score was associated with better DCA curve than module without IRS score. To further validate the prognostic value of the IRS, we compared the prognostic value of each factor which built the nomogram. The Figure 7E shown that the IRS score had highest AUC value 0.726. Multiparameter ROC analysis was also used to calculate the AUC of the nomogram with or without the IRS score. As shown in Figures 7F,G, the AUC of the nomogram was obviously increased from 0.811 to 0.845 after adding the IRS score into the multiparameter ROC analysis, which identified the prognostic value of the IRS score.

## Discussion

KIRC is an inflammation-related carcinoma with a poor therapeutic outcome. Although surgery can improve the prognosis of patients with early-stage KIRC, 20%–40% of patients will still experience recurrence (Liang, 2020). Furthermore, a previous study reported that cancer cell-intrinsic inflammation can facilitate both KIRC metastasis and the initial progression of KIRC (Nishida et al., 2020). To improve the prognosis of patients with KIRC, many inflammatory genes and cytokines were studied to assess their prognostic and curative value. For example, Yang et al. (2021) identified interferon-induced transmembrane protein 2, an inflammation-related gene, as being associated with lymphatic metastasis and poor clinical outcome of KIRC. Another example of this was interleukin-6 (IL-6), an inflammation-related cytokine that could be an independent early-stage immunologic prognostic factor for KIRC patients. The significant role of inflammation in KIRC enlightened us to construct an IRS to enhance the treatment schedule and prognosis of patients with KIRC. The current study selected four differentially expressed inflammation-related genes (TIMP1, PLAUR, CCL22, and IL15RA) and created an IRS based on the four genes. In further analysis, the AUC of the IRS was 0.809, 0.708, and 0.720 at 1, 3, and 6 years, respectively. Furthermore, the multiparameter ROC analysis illustrated that the AUC increased from 0.785 to 0.829 after adding the IRS score to the analysis. Similarly, the prognostic value was identified in the validation groups, suggesting that the IRS could be considered a potential biomarker for KIRC.

There is a close correlation between inflammation-related genes and the occurrence and progression of KIRC. Therefore, we would like to introduce the role of the four genes in KIRC. TIMP1, a key tissue inhibitor of metalloproteinase that regulates most matrix metalloproteinases, was usually found to be increased in renal cell carcinoma (RCC) and could affect the efficiency of radiotherapy (Smyth et al., 2007). Moreover, TIMP1 could promote the invasion and metastasis of RCC and predict the prognosis of RCC (Peña et al., 2010; Lu et al., 2014; Feng et al., 2019). PLAUR belongs to the plasminogen activation system and is widely involved in various cancer-specific processes, including inflammation- and immune- and hypoxia-related pathways (Liu et al., 2021). In addition, several studies have identified that PLAUR is associated with the prognosis of patients with RCC using an online database (Shen et al., 2020; Li et al., 2021). Jin et al. (2019) reported that CCL22 was overexpressed in RCC and could promote the progression and metastasis of RCC by downregulating miR-34a-5p. Furthermore, a study found that CCR4, the chemokine receptor for CCL2 and expressed on T cells, could be considered a therapeutic target for cancer immunotherapy (Yoshie, 2021). Interestingly, in the current study, the infiltration of T cells, including CD8^+^ T cells, gamma and delta regulatory T cells and activated CD4^+^ memory T cells, was higher in the high-risk score group, which suggested that the high-risk score group might be sensitive to immunotherapy. Although few studies have reported IL15RA in RCC, it could predict the prognosis of some cancers and play a protective role in the progression and treatment of some cancers, including colorectal carcinoma, breast carcinoma and multiple myeloma (Marra et al., 2014; Borrelli et al., 2018; Yang et al., 2019; De Mattia et al., 2021). The mechanism of this phenomenon might be caused by upregulation of IL15RA, which could induce the proliferation and activation of NK cells and activate peripheral blood mononuclear cells upon coculture in a paracrine signaling manner (Marra et al., 2014; Borrelli et al., 2018). In the present study, NK cell infiltration in the low-risk-score group was significantly higher than that in the high-risk-score group, which might be one reason why patients in the low-risk-score group had better OS.

To identify the correlation between the IRS and clinicopathological features, we compared the IRS score in different subgroups. The results indicated that high risk scores were associated with T3-4 stage, AJCC stage III-IV and M1, which was also found in the validation group, except for the M stage of GSE29609. A reasonable explanation was the limited number of patients in GSE29609. Moreover, Kaplan-Meier analysis identified that the IRS score could distinguish the prognosis of patients in the male, female, T1-2, AJCC stage I-II and M0 subgroups, and these results also appeared in the TCGA test. Unfortunately, GSE29609 did not perform subgroup analysis due to the limited samples. Furthermore, the IRS clearly differed the patients in the high- and low-risk score groups in all groups. Therefore, we successfully identified the stability and universality of the IRS.

To explore the related pathways, GSVA was used to determine the enriched KEGG pathways by comparing the transcriptome data of the high- and low-risk score groups. The enrichment pathways included primary immunodeficiency, antigen processing and presentation, allograft rejection and systemic lupus erythematosus, which were mainly correlated with inflammation and immune pathways. In accordance with the present results, Zhang et al. (2021) also constructed an inflammation-related signature for gastric carcinoma, and the enriched KEGG pathways were associated with immune pathways. According to the results of tumor mutational burden analysis, VHL and PBRM1 made up the majority of the mutations. Gong et al. (022) reported that VHL gene expression can significantly inhibit the proliferation ability of RCC and promote its apoptosis. However, the high mutation of VHL suggested that it might be a therapeutic target in KIRC. Furthermore, immune checkpoints are widely focused biomarkers of immunotherapy. PD-L1 and HAVCR2 were significantly higher in the low-risk-score group, which suggested that patients in the low-risk-score group might be sensitive to PD-L1-related immunotherapy. As mentioned in the literature review, previous studies reported that many cancers with high TMB scores were usually associated with better survival after receiving immunotherapy (Valero et al., 2021). However, this outcome was contrary to the effect of TMB in RCC, which suggested that a high TMB score could not bring survival benefit to patients who received immunotherapy (Wood et al., 2020). Similarly, the high-risk score group was correlated with higher TMB but was associated with lower expression of PD-L1 and HAVCR2 in this study. Moreover, in our study, patients with low-risk score were associated with lower TIDE score than patients with high-risk score, which might predict a poor response of patients with high-risk score to anti-PD1 or anti-CTLA4 immunotherapy (Jiang et al., 2018). Therefore, compared with patients in the low-risk score group, those in the high-risk score group might benefit less from immunotherapy with the PD-L1 and HAVCR2 immune checkpoints. However, the high-risk score group was positively correlated with the immune infiltration of CD8^+^ T cells, activated CD4^+^ memory T cells, gamma and delta regulatory T cells and macrophages. Therefore, combining the results of TMB, immune checkpoint and immune infiltration, these results might be interpreted cautiously by patients in the high-risk score group, as they might benefit from other immune checkpoints, such as LAG3, rather than PD-L1. Of course, this explanation requires further experiments and studies. Regardless of the explanation, the results of the present study suggest that the IRS is a robust biomarker to predict outcomes and treatment responses in KIRC patients.

This study first identified an inflammation-related signature in KIRC, and we demonstrated its value. It has the potential to become a powerful tool in the management of KIRC patients in clinical practice.

## Conclusion

We found that the IRS might serve as a biomarker to predict the survival of KIRC. Moreover, patients with high or low risk scores might be sensitive to immune drugs at different immune checkpoints.

## Data Availability

Raw data used in this paper were obtained from the public database, and further inquiries can be directed to the corresponding author.
